# Natural compounds as inhibitors of transthyretin amyloidosis and neuroprotective agents: analysis of structural data for future drug design

**DOI:** 10.1080/14756366.2020.1760262

**Published:** 2020-05-18

**Authors:** Lidia Ciccone, Nicoló Tonali, Susanna Nencetti, Elisabetta Orlandini

**Affiliations:** a Department of Pharmacy, University of Pisa, Pisa, Italy; b CNRS, Université Paris-Saclay, Châtenay-Malabry, France; c Interdepartmental Research Centre “Nutraceuticals and Food for Health (NUTRAFOOD), University of Pisa, Pisa, Italy; d Department of Earth Sciences, University of Pisa, Pisa, Italy; e Research Center “E. Piaggio”, University of Pisa, Pisa, Italy

**Keywords:** Natural compounds, transthyretin amyloid diseases, neuroprotection, X-ray structure analysis, drug discovery

## Abstract

Natural compounds, such as plant and fruit extracts have shown neuroprotective effect against neurodegenerative diseases. It has been reported that several natural compounds binding to transthyretin (TTR) can be useful in amyloidosis prevention. TTR is a transporter protein that under physiological condition carries thyroxine (T_4_) and retinol in plasma and in cerebrospinal fluid (CSF); it also has a neuroprotective role against Alzheimer’s disease (AD). However, TTR also is an amyloidogenic protein responsible for familial amyloid polyneuropathy (FAP) and familial amyloid cardiomyopathy (FAC). The TTR amyloidogenic potential is speeded up by several point mutations. One therapeutic strategy against TTR amyloidosis is the stabilisation of the native tetramer by natural compounds and small molecules. In this review, we examine the natural products that, starting from 2012 to present, have been studied as a stabiliser of TTR tetramer. In particular, we discussed the chemical and structural features which will be helpful for future drug design of new TTR stabilisers.

## Introduction

In the last decades, several studies have confirmed that regular consumption of fruits and vegetables, rich in natural substances, can reduce the incidence of different pathologies^1^ including neurodegenerative^2^^,^^3^, cancer and heart diseases, chronic inflammation, and arthritis^4^^,^^5^. Due to all these therapeutic applications, natural compounds are common claimed nutraceuticals and, starting from their chemical structures, they were grouped in different class: flavonoid polyphenols, non-flavonoid polyphenols, phenolic acids, phenolic diterpenes, and organosulphur compounds^6^^,^^7^.

Among these, polyphenols hold one of the largest groups of plant metabolites. More than 8000 polyphenolic compounds have been identified in various plant species. Based on the number of phenol rings that they contain, and the structural elements that bind these rings to one another, polyphenols have been classified into several groups. The main classes include: phenolic acids, flavonoids, stilbenes, and lignans^4^^,^^8^.

Natural compounds have strong antioxidant properties that are often associated to their neuroprotective effects against several neurodegenerative diseases, such as Alzheimer’s^9^^,^^10^, Parkinson’s^11^^,^^12^, Huntington’s diseases, amyotrophic lateral sclerosis, and multiple sclerosis^13^. Various studies have shown that certain polyphenols are able to inhibit the self-assembly of specific peptides and proteins associated with amyloid diseases^14^^,^^15^. Amyloid fibril aggregation is involved in several human degenerative pathologies in which a protein starts to form dimers and small oligomers stimulating the growth of protofibrils and fibrils^16^ that are abnormally deposited in tissue and organs.

More than 40 severe degenerative disorders associated to at least 30 human proteins have been inserted in a group of pathologies called amyloidosis, and transthyretin (TTR) represents one of them. TTR is a ß-sheet rich homo-tetrameric protein characterise by four subunits of 14 KDa each. TTR is synthesised mainly by the liver and the choroid plexus of the brain^17^, in minor amounts in the retina^18^, and in human placenta^19^, therefore, it is present both in human plasma and in the cerebrospinal fluid (CSF), even if at different concentrations^20^.

The acronym TTR suggests the protein’s functions: transporter, thyroxine (T_4_), and retinol. TTR is the main carrier of T_4_ in CSF and the second main carrier in blood^20^. Moreover, TTR tetramer forms a macromolecular complex with retinol-binding-protein (RBP) in which vitamin A binds in a site orthogonal to T_4_^21^. TTR also has a neuroprotective role against Alzheimer’s disease (AD)^22^ taking part in Aβ clearance^23^^,^^24^. Under pathological condition, the concentration of Cu^2+^ and others metals drastically increases in plaque amyloid deposits^25^^,^^26^. Recently, *in vitro* experiments have shown that TTR in the presence of some metals in particular Cu^2+^ and Fe^2+^undergoes a conformational change^27^^,^^28^. Binding between TTR and Aβ peptides with and without copper was performed by bio-layer interferometry and, in presence of Cu^2+^the TTR-Aβ binding affinity increases from micro to nanomolar range^28^.

The TTR tetramer presents a molecular 222 symmetry that generate two identical funnel-shaped named thyroxine binding sites (T_4_-BS), located at a dimer–dimer interface^29^. The T_4_ binding site has a small inner and a larger outer binding subsite. Moreover, in each active site three pairs of symmetric hydrophobic depressions are present. The latter are named halogen binding pockets (HBP1/HBP1′, HBP2/HBP2′, and HBP3/3′) because they are occupied by the iodine atoms of the natural ligand (T_4_)^30^. Even if the two pockets are symmetric, the binding affinity of T_4_ in solution showed that the K_1_ and K_2_ association constants, for the first and the second T_4_ molecule binding to TTR, differ by a factor of about 100, due to a negative cooperativity (NC)^31^^,^^32^. The X-ray structural analysis confirmed the NC binding mechanism showing that, when T_4_ binds to TTR, the two TTR BS diverge in their diameter. The T_4_ binds in the first T_4_-BS, inducing a slight collapse of this site, and simultaneously the opening of the second one. This event is than followed by the binding of the second molecule and the successive collapse of the second site^32^.

Due to high β-strands content TTR is intrinsically amyloidogenic. Under physiological condition, wt-TTR circulates as a soluble protein, but in some elderly or, in patients with TTR single point mutations, it is able to form amyloid fibrils, responsible of neurotoxicity and organ dysfunction. Senile systemic amyloidosis (SSA) is related to wt-TTR deposition and it is has be estimated that ∼25% of individuals over the age of 80 may be affected with SSA^33^. TTR variants^34^ are responsible for more aggressive hereditary TTR amyloidosis (ATTR) in which heart and peripheral nervous system are largely compromised, as in familial Amyloid cardiomyopathy (FAC) and familial amyloid polyneuropathy (FAP), respectively^35^^,^^36^. The most spread mutations are Val30Met which is associated to FAP, Val122Ile that leads to FAC, and Asp18Gly and Ala23Thr which are involved in some central nervous system damaging^37^^,^^38^. Native TTR is more stable and less prone to dissociation compare to TTR mutants in which the tetramer stability decreases, with the exception of some point mutations that are considered non-amyloidogenic variants^34^.

**Figure 1.**
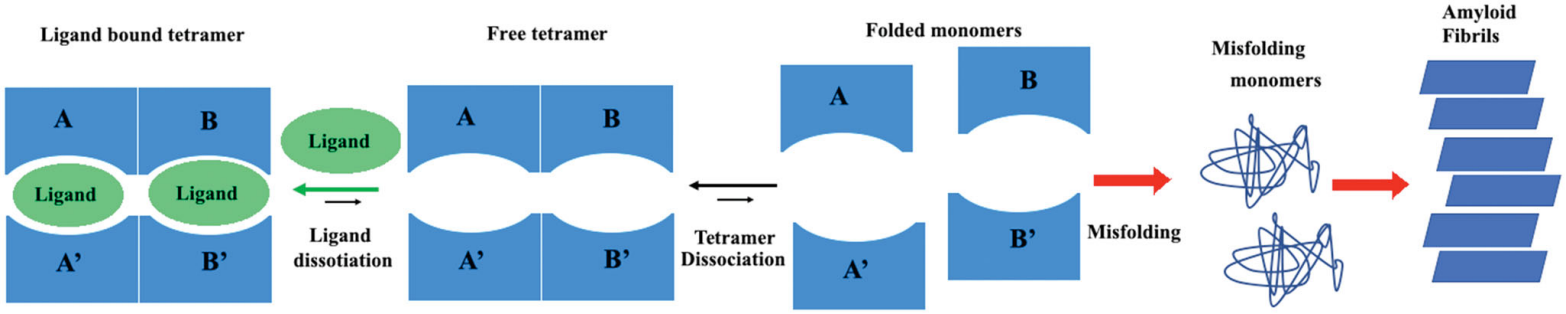
Graphic representation of TTR aggregation pathway.

One therapeutic strategy against ATTR is the stabilisation of the TTR tetramer by small molecules which bind the T_4_ binding pockets preventing the early stage of tetramer dissociation, the first step in the amyloid aggregation process in Figure 1.

This is a reasonable approach because only a small amount of T_4_, in the plasma and in CSF, is bound to TTR, while the majority of T_4_ BS is unoccupied (90–85%). Usually, the majority of TTR inhibitors bind in forward binding mode as T_4_, where anionic substituents, such as carboxylate, are positioned in the outer binding pocket, engaging in electrostatic interaction with the Lys15 and Lys15′. However, other inhibitors can bind to TTR in a reverse binding mode, with the carboxylate oriented towards the inner binding pocket to establish a hydrogen bond (HB) with Ser117 and Ser117′^39^.

In the last two decades, several structurally different small molecules and natural compounds, able to stabilise the TTR tetramer protecting against the fibril formation, have been identified^17^^,^^40^. Randomised controlled trials showed the efficacy of orally administration of TTR stabilisers, such as tafamidis^41^ and diflunisal^42^, to patients in early stage of ATTR amyloidosis^43^. In the last ten years, tafamidis was shown to be effective for cardiomyopathy in ATTR amyloidosis, including wt-ATTR amyloidosis. In 2011, tafamidis was approved by the European Commission for the treatment of ATTR^44^. On 3 May 2019, the US Food and Drug Administration (FDA) announced approval of tafamidis as the first treatments for cardiomyopathy caused by transthyretin amyloidosis (ATTR-CM)^45^.

In 2012, hundreds of small chemical molecules that bound TTR had already been discovered. S. K. Palaninathan published a very exhaustive structural review in which he analysed TTR at atomic level, underlining its biological function and misfolding^46^. In parallel, some of us examined all natural and chemical ligands, belonged to different chemical classes, able to stabilise the TTR tetramer, highlighting the structural modifications that have led to an improvement or to a decrease of their potency and/or selectivity^17^. Since that time, several other natural compounds have been investigated for their ability to inhibit the TTR fibril formation and they have been also co-crystallised with TTR, growing the knowledge about interaction and affinity binding. In Table 1, we have listed all the X-ray crystal structures, deposited at the PDB data bank, in which TTR is in complex with natural products or their metabolites.

**Table 1.** PDB list of all structures in which TTR is in complex with natural compound.

Compound	Mutation	Complex			PDB id	Resolution (Å)
Luteolin						
	wt	TTR-LUT			4DEW^47^	1.90
	wt	TTR-LUT			4QXV^48^	1.12
	V30M	TTR-LUT			4QYA^48^	1.70
	wt	TTR-(7-Cl-LUT)			5EN3^49^	1.25
	wt	TTR-(7-OCH_3_-LUT)			5IHH^49^	1.35
Apigenin						
	wt	TTR-API			4WO0^50^	1.34
	wt	TTR-API			4DER^47^	1.90
Genistein						
	wt	TTR-GEN			3KGU^51^	1.85
	V30M	TTR-GEN			3KGT^51^	1.95
	wt	TTR-(7-O-glucoronide-GEN)			5AKV^52^	1.52
Daidzein						
	wt	TTR-7-O-glucuronide			5AL8^52^	1.50
Quercetin						
	wt	TTR-QUE			4WNJ^50^	1.39
Pterostilbene						
	wt	TTR- Pterostilbene			4WNS^50^	1.39
Naringenin						
	wt	wt			4DEU^47^	1.59
Kaempferol						
	wt	TTR-KAE			4DET^47^	2.05
Chrysin						
	wt	TTR-CHR			4DES^47^	1.75
EGCG						
	wt	TTR-EGCG			3NG5^53^	1.70
γ-Mangostin						
	V30M	TTR-γM			4Y9E^54^	1.49
	V30M	TTR-γM (+ bromine)			4Y9F^54^	1.50
α-Mangostin						
	V30M	TTR-αM			4Y9B^54^	1.40
	V30M	TTR-αM (+ bromine)			4Y9C^54^	1.49
3-Isomangostin						
	V30M	TTR-isoM			4Y9G^54^	1.89
Glabridin						
	wt	TTR-GLA			4N86^55^	2.00
	V30M	TTR-GLA			4N87^55^	1.79
Caffeic acid ethyl ester						
	V30M	TTR- Caffeic acid ethyl ester			4PWG^56^	1.78
Caffeic acid phenethyl ester						
	V30M	TTR-CAPE			4QRF^56^	1.80
Caffeic acid 1,1-dimethylallyl ester						
	V30M	TTR-Caffeic acid 1,1-dimethylallyl ester			4PWH^56^	1.78
Dihydroguaiareti acid						
	V30M	TTR-Dihydroguaiaretic acid			4PWK^56^	1.59
Ferulic acid phenethyl ester						
	V30M	TTR-ferulic acid phenethyl ester			4PWF^56^	1.6
Rosmarinic acid						
	V30M	TTR-Rosmarinic acid			4PWI^56^	1.49
Nordihydroguaiaretic acid						
	V30M	TTR-NDGA			4PWJ^56^	1.55
Retinoic acid						
	wt	TTR-Retinoic acid			1TYR^57^	1.8
Resveratrol						
	wt	TTR-RES			1DVS^58^	1.54
	wt	TTR-RES-T_4_			5CR1^52^	
	wt	TTR-RES-3-O-glucuronide			5AKS^52^	1.25
	wt	TTR-RES-4′-O-glucuronide			5AKT^52^	1.35
	wt	TTR-RES-3-O-sulphate			5AL0^52^	1.39
Curcumin						
	wt	TTR-CUR			4PMF^59^	1.35
Curcumin and ferulic acid						
	wt	TTR-CUR			4PME^59^	1.35
4-hydroxy-chalcone						
	wt	TTR-4-hydroxy-chalcone			5EZP^60^	2.5

This review has the objective to summarises and discuss the literature of all the natural products, published over the last 8 years, that have been studied for their capability to bind and to stabilise the TTR tetramer preventing the fibril formation. For each class of natural compounds, we have reported a detailed chemical and structural analysis in order to investigate if, starting by the observation of natural compounds interactions, we can outline a structure–activity relationship (SAR) for future drug design.

## Flavonoids

**Figure 2.**
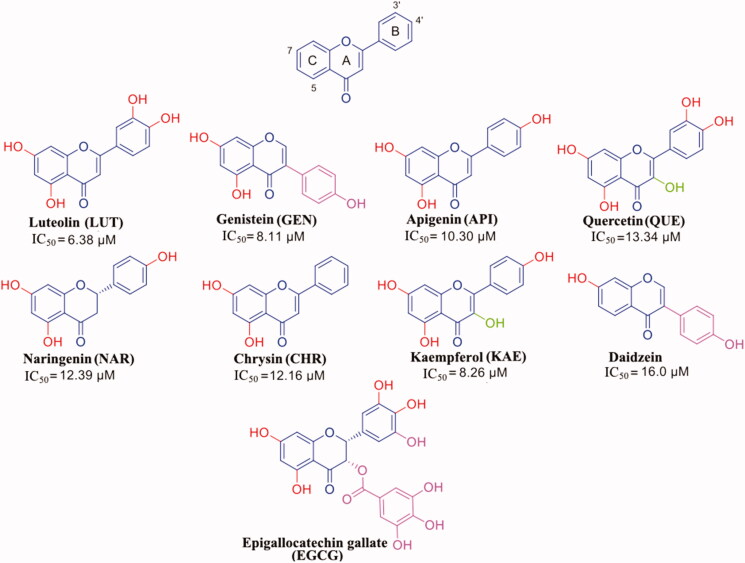
Flavonoids: general formula of the flavonoids selected in this review.

Flavonoids represent a class of natural antioxidant compounds^61^ (Figure 2) that, in the last years, have also been investigated for their neuroprotective effects against different neurodegenerative diseases^62^^,^^63^. Numerous studies showed that flavonoids have variable ability to fit in the two TTR thyroxine binding sites (T_4_-BS) and to inhibit the TTR amyloidogenesis, by stabilisation of the TTR tetramer^47^. Several crystal structures of wt-TTR in complex with them have been reported in the literature, enabling researchers to study both TTR interactions with these flavonoids and the impact of the number and position of hydroxyl groups over the ligand affinity. The results obtained by the crystal structure analysis are often combined with different physical-chemical evaluations either *in vitro* or *in vivo*, giving the possibility to perform some structure-activity relationship study. The determination of the anti-aggregative activity during TTR acid mediated aggregation assays is performed with turbidity measurements, while the estimation of the binding affinities between the drugs and TTR is based on either the classical isothermal titration calorimetry (ITC) or the microscale thermophoresis (MST).

**Luteolin** (**LUT**) is commonly found in spices (parsley, thyme, green pepper, and rosemary), and vegetables (broccoli and carrots). *In vitro* and *in vivo* studies have showed that LUT is a neuroprotective agent against several neurodegenerative diseases: epilepsy, autism spectrum disorders, AD, Parkinson’s disease, traumatic brain injury, diabetes-associated cognitive decline, and multiple sclerosis^64^. Moreover, LUT is known to be the best flavonoid inhibitor of TTR fibril formation (IC_50_ of 6.38 µM)^47^. LUT showed to be able to suppress TTR-induced toxicity in neuroblastoma cells in a cell-based viability assay with a comparable effect as diflunisal and to mediate its stabilising effect on TTR *in vivo* in an established *D. melanogaster* model of FAP. Its ability to prevent the TTR aggregation was assessed by turbidity measurement using a well-established assay where the stability of TTR is monitored at low pH.

**Figure 3.**
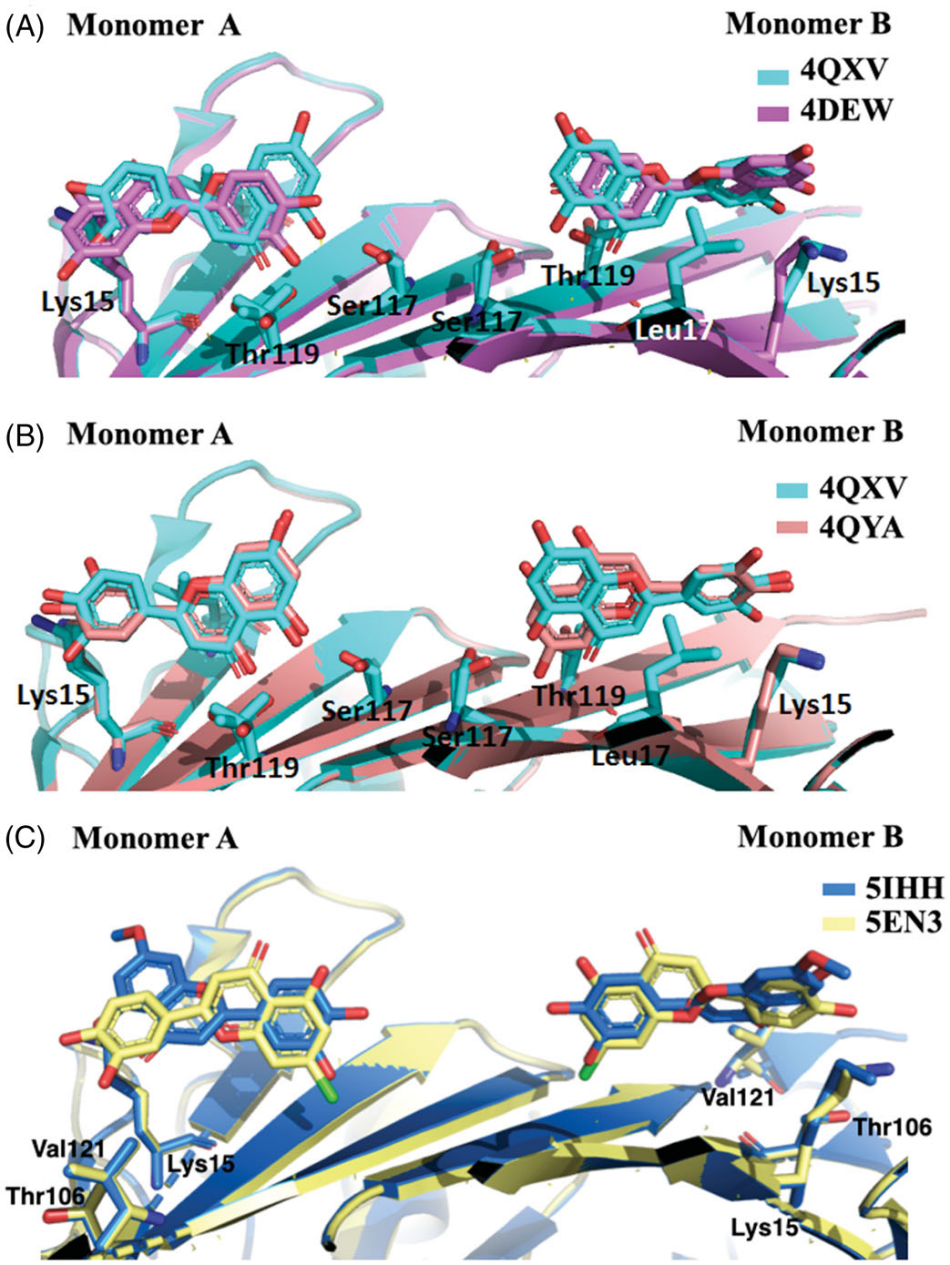
Comparison of the five LUT-TTR crystal structures. (**A)** Superimposition of 4QXV and 4DEV, the two crystal structures of wt-TTR in complex with LUT. (**B)** Comparison between wt-TTR-LUT (4QXY) *versus* V30M-TTR-LUT (4QYA). (**C)** Superposition of two LUT derivatives in complex with TTR.

The most recent crystal structure described by Iakovleva et al. (4QXV) put in evidence the importance of its hydroxyl groups at position 5 and 7 (ring AC) and the simultaneous HBs over the tetramer interface^48^. The comparison of the previously reported TTR-LUT structures (4DEW) with the high resolution complex 4QXV suggests that, to correctly fit into the electron density map, 4QXW should be rotated 180°^50^ (Figure 3(A)). The two hydroxyl groups are oriented towards the side chains of Ser117 and Thr119 and the symmetry-related copies of the same residues over the dimer–dimer interface (AA’ and BB’ sites). The ring B is involved in the contact with the side chain of Leu17 and the Cδ and Cε atoms of Lys15 by van der Waal interactions, without direct HBs between the two hydroxyl groups in 3′ and 4′ positions and the N_Z_ of the Lys15. The crystal structure of V30M-TTR in complex with LUT is structurally very similar to wt-TTR-LUT^48^ (Figure 3(B)).

LUT poorly explore the centre of the wt-TTR binding cavity, which is the narrowest portion of the wt-TTR binding site, and establish polar contacts at the entrance and bottom of T_4_-BS. As deducted from ITC experiments, this type of interaction is able to give the best inhibition by acting with an “identical and independent” mechanism of binding with the two sites of wt-TTR.

In humans, LUT is metabolised by liver and intestines in the 7-position leading to inactive derivatives. To prevent the glucuronidation, two analogues, in which the hydroxyl group at the 7-position was replaced by chlorine atom (7-Cl-LUT) or a methoxyl group (7-MeO-LUT), were synthesised^49^. The MST binding-assay showed that both 7-Cl-LUT and 7-MeO-LUT have lost some of their binding affinity compared to the LUT structure (K_d_ values of 620 ± 150 and 390 ± 40 nM, respectively, compared to LUT 150 ± 70 nM), but they still demonstrated to be highly strong binders for TTR, with the methoxy substitution more tolerated than the halogen one. The TTR-(7-Cl-LUT) crystal complex (5EN3, Table 1) showed only minor differences in protein-ligand interactions compared to the TTR-LUT structure. If the orientation of ring B and the OH groups in 3′ and 4′ positions are the same, the flavone ring is horizontally flipped, thus allowing only the HB of the hydroxyl group in 5 with Ser117 and Thr119 only with one subunit. At the contrary, TTR-(MeO-LUT) crystal complex (5IHH, Table 1) displayed a reverse binding mode in which the methoxy group is placed into the hydrophobic pockets, reached by Lys15, Thr106 and Val121, and the hydroxyl in position 4′ points towards the inner cavity (Figure 3(C)), and establishes HBs with both Ser117 of the two A and A’ subunits and the Thr119 of subunit A’^49^.

Taking together, these results put in evidence the importance of the OH group in 7-position for the interaction with the T_4_-BS. Its substitution is quite tolerated by a methoxy group which reverses the contact orientation of the molecule in the binding pocket but maintains a similar interaction network.

**Genistein** (GEN) is an isoflavone extracted from soy bean and described as an angiogenesis inhibitor and a phytoestrogen. GEN is also known for its antioxidant properties, its cardioprotective, chemoprotective, and neuroprotective effects^65^^,^^66^. It was proved that GEN is an oestrogenic isoflavone more selective to oestrogen receptor β than α, thus representing a potential therapeutic candidate against menopausal symptoms^67^. Moreover, different studies showed that GEN binds with high selectivity to TTR, both in plasma and in CSF, and strongly inhibits TTR fibril formation^68^^,^^69^. ITC analysis, performed by Trivella et al., demonstrated that the allosteric perturbations caused by GEN binding to the first wt-TTR binding site are intrinsic in the apoV30M mutant. This mutation causes differences in GEN binding mechanism, binding affinity, and tetramer stability. Acid-mediated dissociation/denaturation assays showed that the protective effect of GEN on the wt-TTR aggregation is more evident than that observed for V30M (IC_50_=6.7 µM for wt-TTR and 9.7 µM for V30M). This result was further confirmed by another biophysical technique assessing the tetramer stability by using high hydrostatic pressure (HHP). HHP is a perturbing tool in which the tryptophan emission is used as a sensor of tetramer dissociation. Overall these findings show that TTR mutants may present different ligand recognition and therefore extremely important during ligand design for achieving the best inhibition activity^51^.

**Figure 4.**
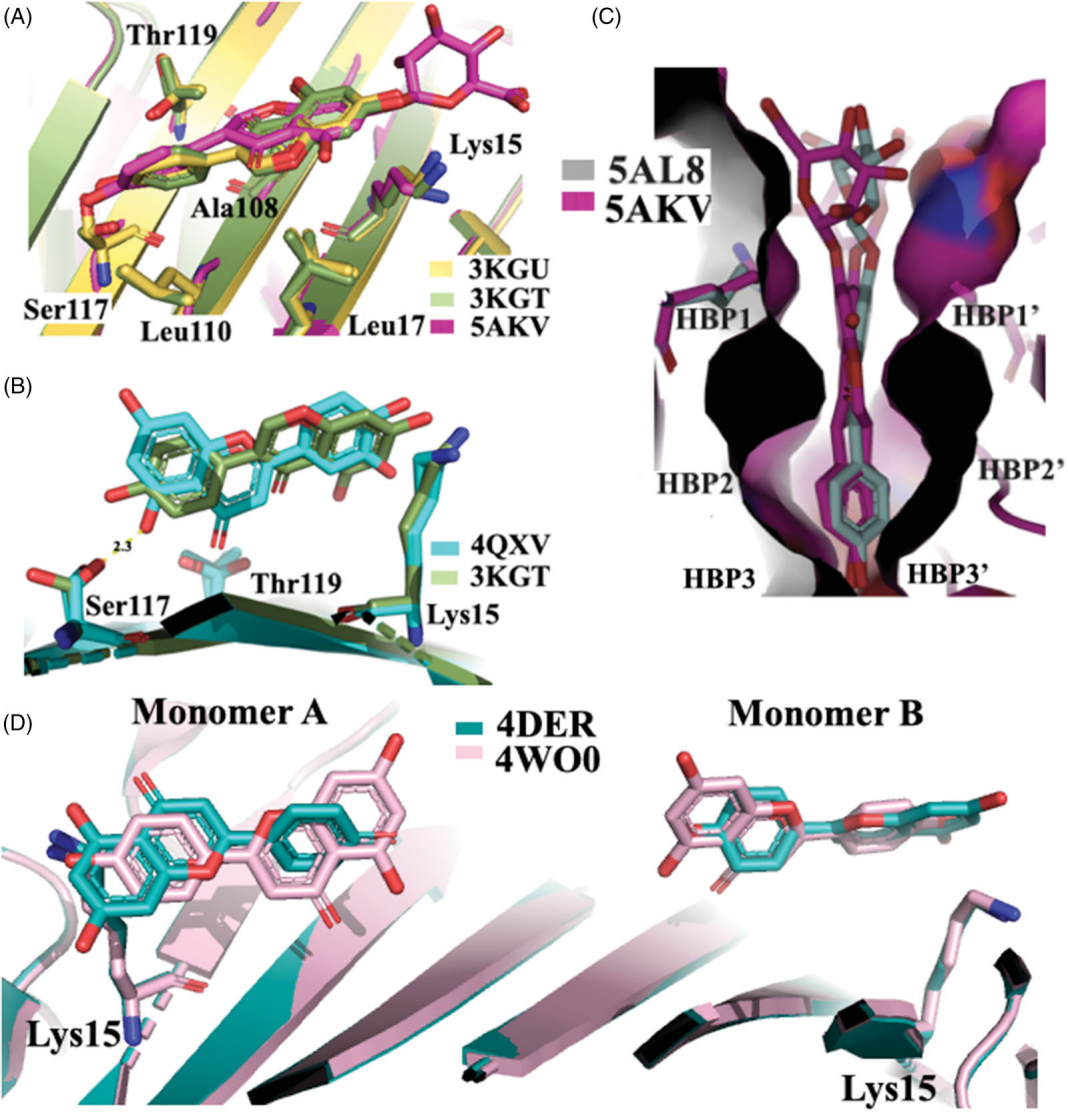
Comparison of isoflavones derivatives and their metabolites. (**A)** Superimposition of GEN in complex with wt-TTR (3KGU), GEN in complex with TTR mutant V30M (3KGT), and genistein 7-O glucuronide complexed with wt-TTR (5AKV). (**B)** Structural analysis of TTR-LUT crystal structure (4QXV) and the TTR mutant V30M in complex with GEN (3KGT). (**C)** Comparison between the crystal structures of the two glucuronide metabolites of daidzein (5AL8) and genistein (5AKL) in complex with wt-TTR. (**D)** Superposition of the two TTR-API crystal complexes present in the PDB data bank.

The X-ray crystallographic structures of GEN bound to wt-TTR and V30M were solved (3KGU and 3KGT)^51^. The superposition of the two crystal structures shows that the phenolic ring B is deeply placed into the inner binding pockets with the A and C rings pointed towards the outer cavity and the -OH, in position 7, is oriented towards the Lys15 as shown in Figure 4(A). The hydrophobic portion is contoured by the apolar portion of residues Leu17, Leu110, Lys15, and Ala108. The crystallographic analysis confirms that GEN binds, to wt-TTR and V30M BS following a negative cooperative mechanism^51^^,^^68^. Comparing the two PDB structures, there are not significant differences between the GEN orientation and the amino acid side chain position in the two BS in wt-TTR and V30M-TTR. However, using the biophysical techniques mentioned above and comparing these results with those obtained by X-ray crystallography (apo-TTR, apo-V30M, TTR-GEN, and V30M-GEN), it was proved that just a single point mutation (V30M) could induce dynamic changes in the wt protein which are similar to the allosteric perturbations induced after GEN binding^51^.

The single V30M mutation causes differences in GEN binding mechanism and alters the dose-dependent effects of the GEN-inducted protein stabilisation.

GEN presents a similar affinity as LUT for wt-TTR but not for V30M TTR mutant. The difference of affinities seems not related to the different position of the phenyl ring on the benzopyran moiety and the absence of its OH group in 3′ position (Figure 2). Rather, GEN seems to enter shallower than LUT in the binding pocket and establishing an HB with Ser117 by its OH group in 4′^51^. Ser117 is in a conformation similar to that found in the allosteric movement, involving wt-TTR after the first binding-ligand (negative cooperative mechanism). This different ligand recognition should be taken into consideration for future design of TTR amyloidosis inhibitors.

GEN is a very good inhibitor of TTR amyloidogenesis compared to its glucoside derivative genistin (41% aggregate formation at 36 μM, 10 times TTR plasma concentration) or its 7-O-glucuronide derivative. A plausible explanation could be furnished by the X-ray analysis of TTR-7-O-glucuronide crystal complex (5AKV) in which the poorly defined electron density of the glucuronide function is placed outside the binding pockets (Figure 4(A,C)). It can be assumed that the steric hindrance of the glucuronide group impedes the perfect allocation of the inhibitor in the T_4_ binding pockets^52^.

**Daidzein** is another abundant isoflavone found in soy food which has been reported to have anti-inflammatory^70^, cardioprotective^71^, chemoprotective^72^, and neuroprotective^73^^,^^74^ effects. Daidzein differs from GEN in the lacking of –OH group in position 5 (Figure 2). The absence of hydroxyl group, at position 5, deeply decreases the daidzein efficacy against the aggregation inhibition potency as well as its ability to bind TTR^68^. More recently, its binding affinity (IC_50_=16 µM) was assessed by ThT fluorescence assay in comparison with diflunisal^55^. No significant binding affinity is visible between TTR and daidzein-7-O-glucuronide, the major metabolite found in plasma. As for the GEN 7-O-glucuronide, also for daidzein-7-O-glucuronide the decreased capacity to stabilise the TTR tetramer could be attributed to the steric hindrance of the glucuronide moiety located outside of the HBP1/1′ (Figure 4(C))^52^.

**Apigenin** (**API**) is a flavone presents in fruit and vegetables, especially the ligulate flowers of chamomile plant are rich of API. API has potent antioxidant, anti-inflammatory, and anti-carcinogenic properties, moreover, it is able to cross the brain-blood-barrier and to protect against neurodegenerative disease such as Alzheimer’s^75^^,^^76^ and ATTR^47^.

API has the same structure as LUT except for the lack of the hydroxyl group in position 3′ at the ring B as shown in Figure 2. The absence of this group affects the binding affinity (IC_50_=10.30 µM) and suggests the importance of the presence for the activity of both OH on the ring B of LUT, even if they are not directly involved in any HB with the Lys15.

API proved to have a similar ability as GEN to stabilise the TTR in presence of denaturing urea solution and to inhibit the TTR fibrillogenesis by turbidimetric assay. However, in fluorimetric competition binding assay, API showed to be able, as GEN, to displace resveratrol from its preferential binding site, while in the presence of radiolabeled T_4_ only GEN exhibited the highest binding selectivity. This suggests that the different position of the phenyl B ring in GEN than API can compensate the absence of the OH group in 3′ and modulate a better binding affinity.

The first TTR–API crystal complex (4DER) showed that API bound in a very similar fashion to LUT with a slight difference at the bottom part^47^. In 2015, a new X-ray structure of TTR in complex with API was solved (4WO0)^50^, in this case the benzopyran ring is placed inside the cavity and the hydroxyphenyl ring points towards the outer subsite (Figure 4(D)). The API allocation, in the binding pocket, is opposite to that previous deposited structure 4DER (Figure 4(D)). However, it is not easy to explain the discrepancy between the opposite results obtained both for LUT and API regarding their binding mode. Maybe it can be imputed to the different crystallisation condition, or to the lower resolutions of the older structure.

**Quercetin** (**QUE**) is found in many fruits, vegetables, leaves, and grains; red onions and kale are common foods containing appreciable content of QUE. It is classified as an antioxidant agent, by acting as a scavenger of free radicals. It has been shown *in vitro* its activity as a non-specific protein kinase enzyme inhibitor and agonist of the G-protein coupled oestrogen receptor^77^^,^^78^. QUE is also considered an anticancer and neuroprotective agent^79^^,^^80^. *In vivo* study showed that oral QUE administration, in triple transgenic AD mice model, reduces β-amyloidosis and decreases tauopathy in the hippocampus and amygdala^81^.

The molecular structure of QUE has one more hydroxyl group than LUT on benzopyran ring as in Figure 2. Similarly to the other flavonoids, QUE interacts with TTR even if this simple structural modification affects the binding affinity and its IC_50_ increases of double^47^ compared to LUT (13.34 µM *versus* 6.38µM) (Figure 2). The binding experiments in the presence of radiolabelled ^125^I-T_4_, as a competitor, indicated that QUE does not displace the TTR-bound hormone even at highest concentration (50 µM). On the contrary, fluorescence data showed that QUE is able to induce the displacement of TTR-bound to pterostilbene, which can bind to TTR independently from the presence or the absence of pre-incubated T_4_^50^. These results suggested a different binding site for polyphenol compounds compared to T_4_ hormone, with a superior affinity for benzopyran structure to the stilbene one.

**Figure 5.**
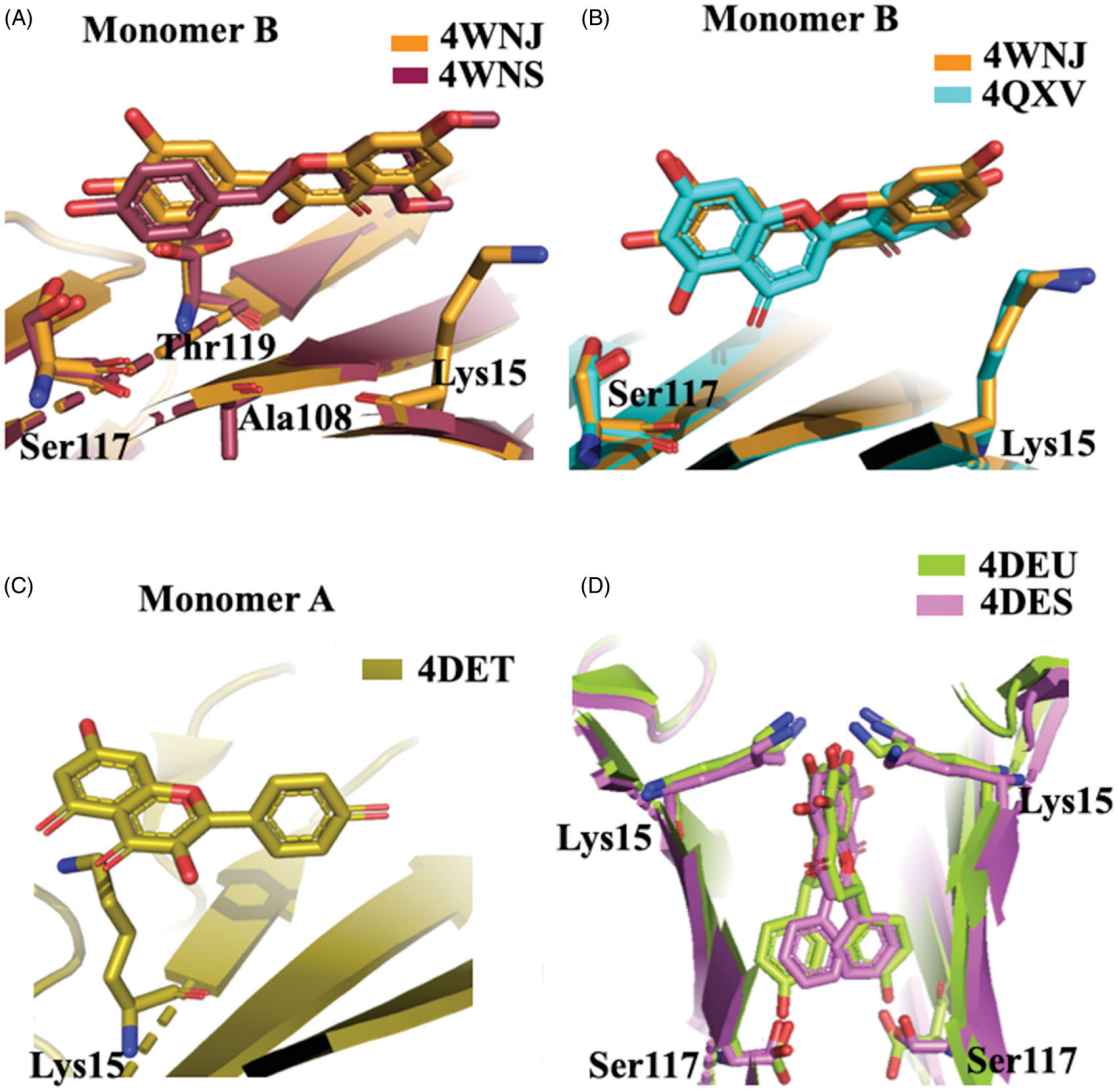
Structural analysis of quercetin (4WNJ), pterostilbene (4WNS), kaempferol (4DET), naringenin (ADEU), and Chrysin (4DES) in complex with TTR. (**A)** Comparison between QUE pterostilbene crystal structures. (**B)** Superposition between QUE and LUT crystal structures. **C** Graphic representation of TTR-KAE crystal complex. (**D)** Superposition between NAR and CHR crystal structures.

The crystal structures of TTR-ligand complexed deposited in the PDB data bank do not furnished an explanation about the difference in the binding mechanism, obtained in solution. The superposition of TTR-QUE (4WNJ) and TTR-pterostilbene (4WNS) structures shows that the ligands fit in T_4_ binding site, with a good density, only with site B using a negative binding cooperativity^50^ (Figure 5(A)). The root-mean-square deviation (r.m.s.d.) calculated on Cα between the two complexes is 0.24 Å, indicating that the structures are very similar^50^. The pterostilbene shows a planar conformation with the (dimethoxy)phenyl groups pointing towards the Lys15 and the phenol rings placed into the inner cavity. QUE fits in the T_4_ pocket orienting the benzopyran ring towards the outer cavity while the (dihydroxy)phenyl ring is stabilised in the inner pocket by the interaction with Ser117 (Figure 5(A)).

The different affinity of QUE compared to LUT (the most active flavonoid) is principally due to the presence of an additional hydroxyl group that establishes a weaker binding with TTR. The presence of a substituent in the 3-position of the benzopyran nucleus, as observed in QUE and GEN, seems to be responsible of the reverse position of the ligand in the binding pocket compared to LUT one as shown in Figure 5(B). However, the reversing of the binding position seems to have less influence upon the ligand affinity to the second binding site, compared to a removed OH from the ring B (API *versus* LUT). The different affinity between QUE and GEN suggests that the hydrophobic substitution in the 3-position is more tolerated than a polar one. An acceptable binding affinity is restored when the OH in 3′ position of the ring B of QUE is removed (see kaempferol (KAE) in Figure 2).

**Kaempferol **(**KAE**) is a natural flavonol found in a variety of plants and plant-derived foods, including grapes, tomatoes, broccoli, tea, and ginkgo biloba leaves (Figure 2). This biologically active compound exhibits many pharmacological properties including antioxidant, anti-inflammatory, antimicrobial, antidiabetic, anticancer, and neuroprotective activities^82^^,^^83^. KAE (IC_50_=8.26 µM)^47^ showed a similar interaction to QUE in the TTR binding pocket, with the AC ring interacting with Lys15 side chains at the entrance of the binding site and with the B ring, devoid of hydroxyl in position 3′, pointing to the bottom of the binding site (Figure 5(C)).

**Naringenin** (**NAR**) and **Chrysin** (**CHR**) are other two flavonoids that interact with TTR. *In vitro* and *in vivo* studies reported that NAR have different pharmacological effects: antioxidant, antitumor, antiviral, antibacterial, anti-inflammatory, antiadipogenic, and cardioprotective^84^. Recently, it has been reported that NAR can be consider promising therapeutic potential for Parkinson’s disease^85^^,^^86^. The neuroprotective effect of NAR is also attributes to its ability to increase the level of neuroglobin, a protein that protects both heart and brain tissues from hypoxic injury^87^.

CHR has protective effects against several pathologies such as cancer, diabetes mellitus, cardiovascular diseases, obesity, and allergic events. Scientific studies have also proved that CHR has neuroprotective and hepatoprotective properties in numerous animal models^84^.

NAR and CHR have similar structure to API. NAR shows a stereocenter at 2 position of the benzopyran nucleus, due to the double bond saturation, while CHR presents a ring B without any hydroxyl group (Figure 2). Both these modifications affect negatively the binding affinity (IC_50_=12.39 and 12.16 µM, respectively) compared to API^47^. The explanation could be related to a higher degree of freedom for the lack of the C ring double bond in the case of NAR and to the unfeasibility to establish polar contacts to the binding site due to the absence of hydroxyl groups in the case of CHR. The comparison between the crystal complexes TTR-CHR and TTR-NAR displays that the two ligands are oriented in T_4_-BS in very similar manner (Figure 5(D). The B ring binds deeply into the inner binding site while A and C pointed towards Lys15.

**Epigallocatechin gallate **(**EGCG**) is the major polyphenol component of green tea (Figure 2). EGCG has several biological functions, it has been studied for its anti-oxidative and anti-inflammatory effects, in addiction *in vitro* experiments have been reported that EGCG is a potent anti-cancer agent^88^. EGCG is considered a viable therapeutic candidate to prevent the progression of neurodegenerative diseases like Alzheimer’s disease, amyotrophic lateral sclerosis, multiple sclerosis, and Parkinson’s disease^89^^,^^90^. Moreover, EGCG has been described to be able to bind to TTR and suppress TTR amyloid fibril formation, by different biophysical assays: *in vitro* dynamic light scattering (DLS) and transmission electron microscopy (TEM), *ex vivo* isoelectric focussing (IEF), and *in vivo* immunohistochemistry (IHC) in mice models. The interaction with TTR has been studied by T_4_ competition assay, ITC, and X-ray, while the stabilisation of the TTR tetramer structure by SDS-PAGE^53^^,^^91^. EGCG proved to bind strongly to the protein and to stabilise its tetramer conformation, thus inhibiting the aggregation *in vitro* and in a cell culture system, and maintaining most of the protein in a non-aggregated soluble form. However, EGCG has a mode of action different from those of the natural compounds described above. Some of us previously discussed the interaction between TTR and EGCG elsewhere^17^ and, in 2019, Ferreira et al. published an interesting review in which is also described the *in vitro* and *in vivo* studies of TTR-EGCG interaction^92^. The analysis of TTR V30M mutant in complex with EGCG showed that EGCG does not bind into the canonic T_4_-BS, usually occupied by the TTR stabilisers, but it binds to three BS located at interface between monomers A and B and their symmetric operations^53^. This interaction, which stabilises not only the tetramer structure but also induces oligomer formation, was proved in CHO-K1 cell culture system.

Therefore, regarding EGCG, we would like to highlight that the three novel BS found, distinct from those of the BS of T_4_, suggest the possibility to explore new target sites for promoting TTR stability and to strengthen the effect of tetramer stabiliser^53^^,^^93^.

**Figure 6.**
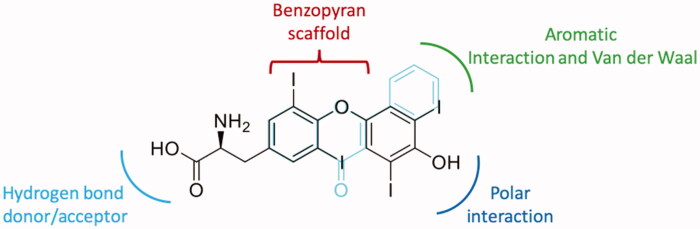
Comparison between flavonoids structure and endogenous T_4_ ligand.

The development of other drugs is of great importance for the treatment of ATTR. Even if tafamidis is effective for cardiomyopathy in wt-ATTR^94^^,^^95^ and halted or slowed disease progression for up to 18 months in patients with early-stage ATTR V30M-FAP^96^^,^^97^, the therapy costs are high and the efficacy is reduced in patient with advanced disease, thereby is important to study newer potent stabilisers^98^. Active compounds isolated from natural sources can be considered hits for future therapeutic innovation and the information obtained from X-ray crystallography is of great importance for establishing the most crucial interactions with the target. With the aim to develop new compounds with greater affinity and better selectivity for the second binding site of the TTR tetramer, the crystallographic results obtained from the TTR-flavonoid interaction studies can help to design new inhibitors. By comparing the structure of flavonoids with the endogenous TTR ligand (T_4_), it is possible to see how a benzopyrane scaffold adapts well to the binding cavity and allows the orientation of the substituents necessary for the interaction (Figure 6). An examination of the chemical structural features of flavonoids and T_4_ allow some interesting considerations:

- On the “right side,” one aromatic and one polar function are necessary to establish Van der Waal and hydrophobic interactions with the inner cavity of the binding pocket.
- On the “left side” acceptor/donor substituents are required to provide hydrogen bonding at the entrance of the cavity.

The whole obtained results allow to define a SAR for flavonoids chemo type: modification and deletion in position 3′ on the B-ring and substitution in position 2 and 3 on the AC-ring are well tolerated, while the double bond and the two hydroxyl groups in 5 and 7 on the AC-ring result very important for the binding affinity.

## Xanthonoids and isoflavane

**Figure 7.**
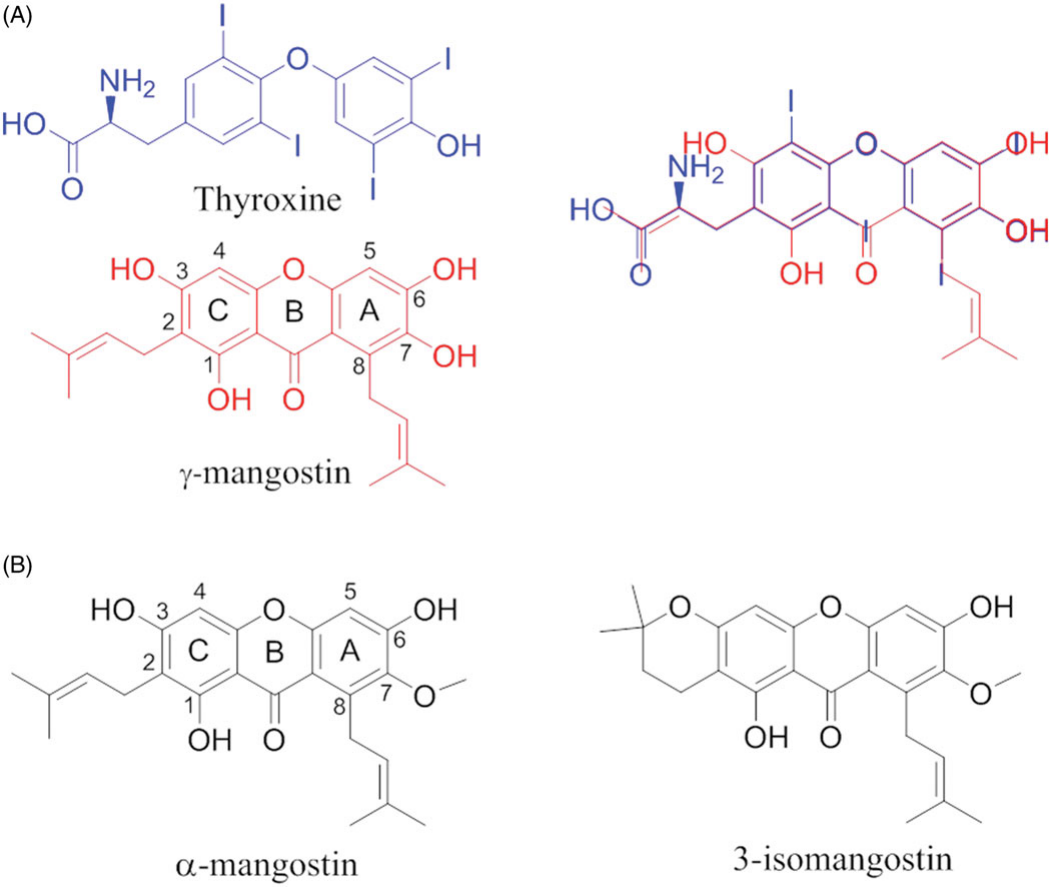
Chemical structure of thyroxine, α, γ and 3-isomangostin and their graphical superposition.

**γ-mangostin** (**γ-M**) is a natural xanthonoid, a type of organic compound isolated from various parts of the mangosteen tree (*Garcinia mangostana*), with a xanthone core structure. γ-M and a variety of other xanthonoids from mangosteen have been investigated for biological properties including antioxidant, anti-bacterial, anti-inflammatory, anticancer, and neuroprotective activities^99^^,^^100^. In 2015, an interesting study about the interaction between TTR and 12 xanthone derivatives was published^54^. The most promising TTR stabilisers were γ-M and α-mangostin (α-M) (Figure 7(A,B)), while the 3-isomangostin (Figure 7(A)), cyclised derivative of α-M, displayed significantly lower capability to inhibit the fibrillisation process^54^.

By comparing the molecular structure of γ-M and T_4_, it is possible to note the similarity of γ-M with the endogenous ligand, in particular, the two aromatic rings and the dimethylallyl group which superpose exactly with the T_4_ structure (Figure 7(A)). At the same time, it is possible to recognise the benzopyran ring typical of flavonoids. This can let hypothesise a similar binding affinity for the TTR. γ-M is an effective inhibitor against the amyloid fibril formation of V30M amyloidogenic TTR. *In vitro* binding assays by fluorescence spectroscopy, pull-down assay using CN-Br activated Sepharose, ANS competitive test, and cross-linking followed by SDS-PAGE, revealed that γ-M is the most potent of the all tested xanthone derivatives (IC_50_=7 µM), and it binds to the T_4_-BS and stabilises the TTR tetramer^54^.

**Figure 8.**
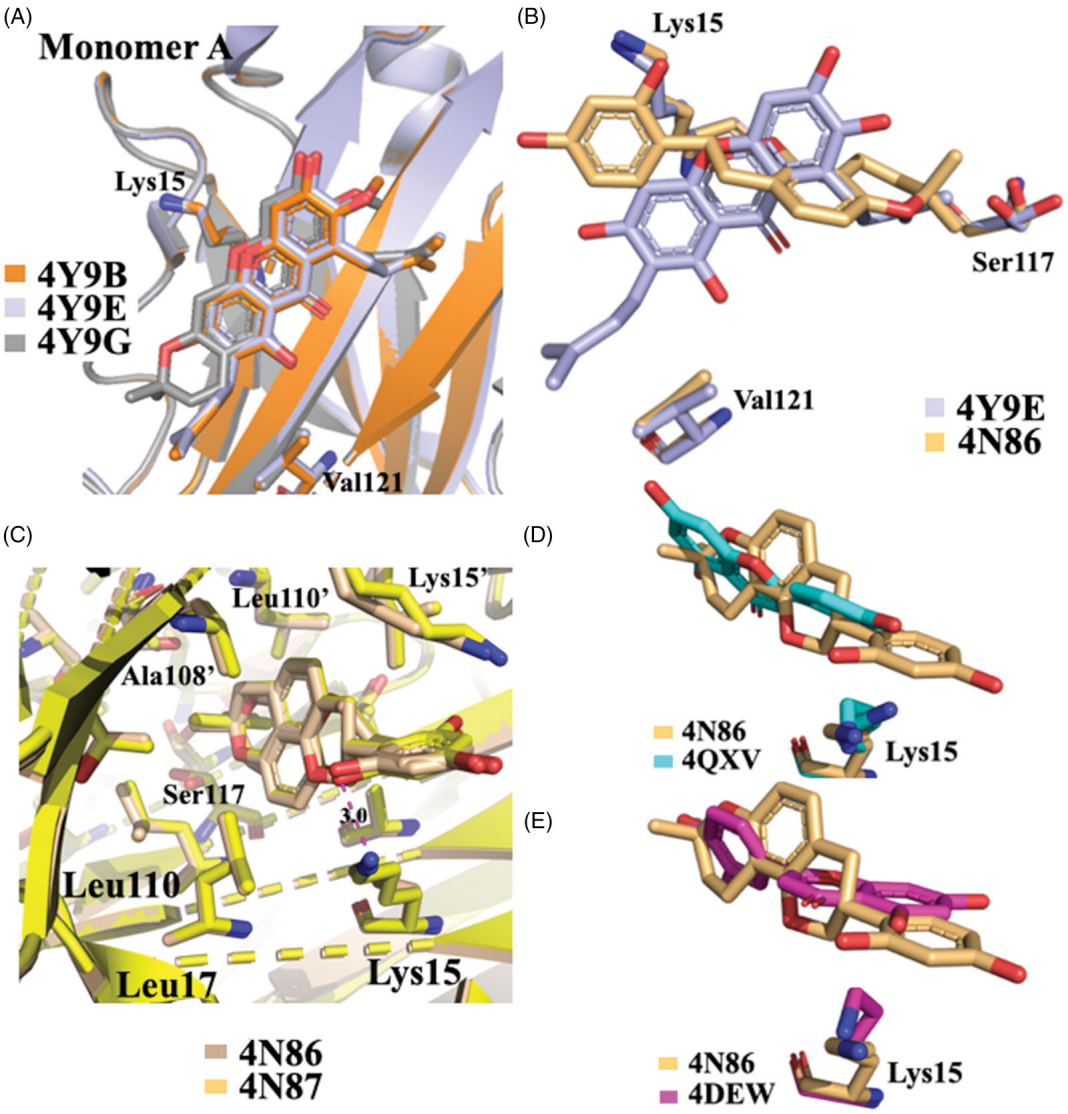
Structural analysis of xanthonoid and isoflavanes derivatives. (**A)** Superposition of V30MTTR-α-M (4Y9B), V30MTTR-γ-M (4Y9E), and V30MTTR-3-isomangostin (4Y9G). (**B)** Diagonal binding mode of γ-M compare to glabridin (4Y9E *versus* 4N86). (**C)** Comparison between TTR-glabridin crystal complex wild type and V30M mutant, 4N86, and 4N87, respectively. (**D** and **E)** Superposition of ligands in wt-TTR-glabridin and the two deposited structures of TTR-LUT deposited in the PDB data bank.

Five different V30M TTR mutant crystal structures were solved in complex with γ-M, α-M, and 3-isomangostin (Table 1). The crystals grown belong to the orthorhombic space group P2_1_2_1_2 with the cell parameters comparable to those deposited in the PDB data base. The resolution of the structures ranges from 1.90 to 1.40 Å. The structural analysis between TTR-γ-M and TTR-α-M crystal complexes showed that the position and the orientation of the xanthones are very similar in T_4_-BS (r.m.s.d. 0.36 Å)^54^. Even so, the mangostins stabilise the TTR tetramer displaying a different binding mode compared to the most known TTR stabilisers (Figure 8(A,B)). The γ-M binds diagonally against the 2-fold axis of the BS at the interface between two subunits of TTR (AA’ or BB’), and its binding is associated with two chloride ions, derived by the crystallisation conditions^54^. The A-ring of γ-M is located at the inner cavity of the T_4_-BS and the C-ring is located at the outer cavity, with the result that 3-OH turned to the solvent. The 2-dimethylallyl group points to the side chain of Val121 of one subunit, while the 6- and 7-OH point to another subunit. The 8-dimethylallyl group is sterically surrounded by hydrophobic groups, confirming the presence of a hydrophobic part of the ligand necessary for hydrophobic contacts in the inner binding pocket. Atomic repulsion because of the presence of an additional OH in the 8-methylallyl group causes lowered inhibitory activity. Methylation of 7-OH in α-M decreases the affinity for TTR, demonstrating the important role of 6- and 7-OH for establishing HB *via* halide ions. Cyclised 2-dimethylallyl derivatives showed the importance of flexible 2-dimetylallyl group interacting with the side chain of Val121 in the outer cavity of the pocket where the 3-OH is implicated in HB with the solvent. The position of the 8-dimethylallyl group is important for the inhibitory activity as well as the position and number of hydroxyl groups, especially for 6- and 7-OH.

**Glabridin** (**GLA**) is a prenylated isoflavane originally isolated from the roots of *Glycyrrhiza glabra L.* (Favaceae), commonly known as liquorice. GLA is the most studied liquorice flavonoids, is considered to be a phytoestrogen and is associated with numerous biological properties, including antioxidant, anti-inflammatory, neuroprotective, antitumorigenic, and skin-whitening activities^101^. Acid-mediated aggregation experiments by a Thioflavin T assay revealed that GLA is a potent amyloid fibril formation inhibitor with an inhibitory activity equal to that of diflunisal (IC_50_=6.4 µM). Comparing the molecular structure of GLA, GEN, and Diadzein, it is possible to recognise a similar AC-ring scaffold.

**Figure 9.**
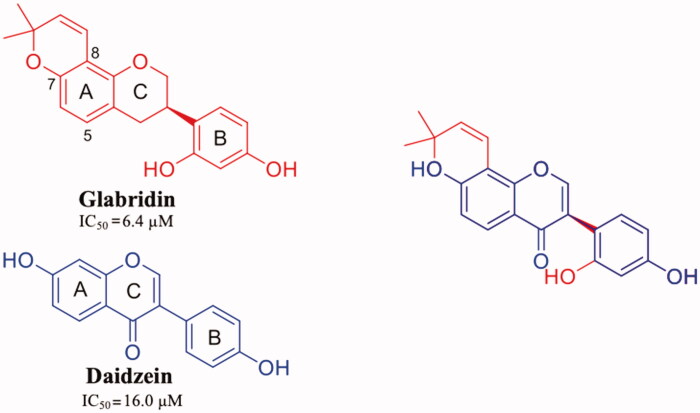
Chemical structure of glabridrin and daidzein, and their graphical superposition.

Despite the absence of the 5-OH and the double bond, as observed for LUT, GLA maintains a potent inhibitory activity thanks to the 8-dimethylallyl group, cyclised with 7-OH of the AC-ring, in the similar way observed for γ-M (ring A) (Figure 9).

GLA was crystallised both in complex with wt-TTR and V30M TTR mutant (4N86 and 4N87, respectively)^55^. There are not significant structural changes comparing the TTR-GLA and V30M-GLA with apo-TTR (Figure 8(C)) (r.m.s.d. of Cα atoms between apo-TTR and TTR-GLA was 0.24 Å and between apo-TTR and V30M-GLA was 0.34 Å . GLA binds to TTR BS similarly to other flavonoids. If we consider the most recent LUT PDB structure (4QXV)^48^ GLA and LUT have the same orientation. On the contrary, by examining the first LUT structures (4DEW)^47^, the orientation of GLA is opposite (Figure 8(D,E)). The B-ring of LUT, in 4QXV crystal structure, is allocated into the inner T_4_-BS, while the B-ring of GLA points towards the outer cavity stabilising its interaction with the HB between 2′-OH and Lys15. The cyclised prenyl group of GLA is well placed into the hydrophobic T_4_-BS and the aromatic A ring shows a CH-π interaction with A108-C^β^^55^. Another effect of the binding between TTR and GLA is the conformational change of Thr119 with the introduction of a water molecule that plays an important role in the tetramer stabilisation. Moreover, this water forms a HB network that induces a conformational change of the Tyr114 side chain which is fundamental for the tetramer stability.

Concluding, when GLA binds to TTR induces several small changes that result into a high stabilisation of tetrameric structure.

## Phenolic acids: caffeic, rosmarinic, and retinoic acids

**Figure 10.**
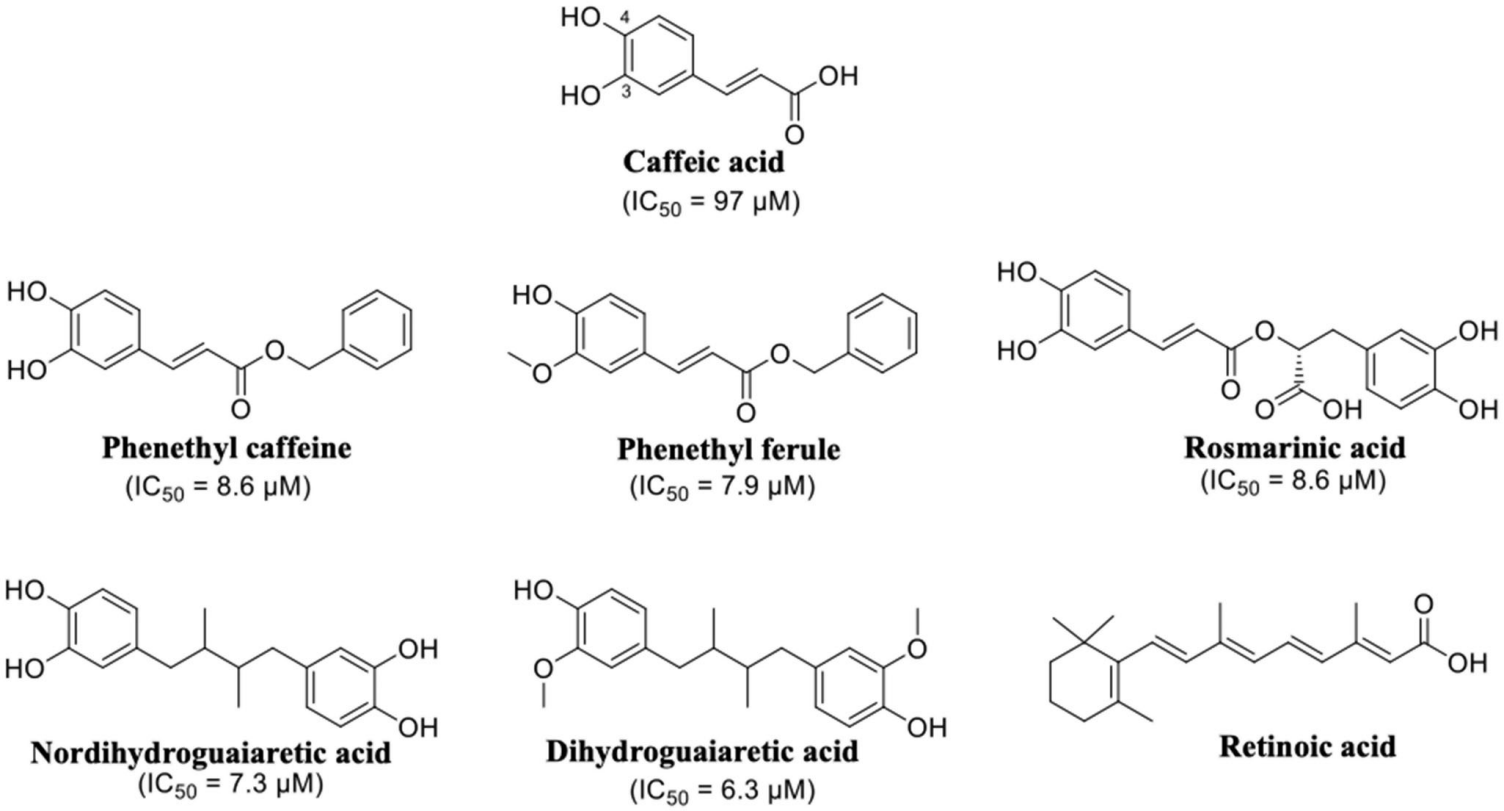
Chemical structures of caffeic acid, its ester derivatives, NDGA, and dihydroguaiaretic acid.

Propolis has been shown to exhibit a broad spectrum of biological activities, such as antioxidant, anti-inflammatory, immunomodulatory, and anti-cancer properties^102^^,^^103^. Propolis and its components have also been investigated for their neuroprotective effects against oxidative stress^104^ and AD^105^^,^^106^. The chemical composition of propolis is variable, but caffeic acid phenetyl ester (CAPE) is one of the most active of the compounds isolated. The structural similarity between caffeic acid, rosmarinic acid, nordihydroguaiaretic acid (NDGA) and the known inhibitors such as diflunisal and RES had let to speculate their possible activity as TTR aggregation inhibitor. In 2011, these compounds were studied as inhibitors of TTR fibril formation, and NDGA resulted the most promising compound^91^. Few years after, in 2014, Yokoyama et al. investigated CAPE, its analogues and several lignans, including NDGA (Figure 10), by ThT fluorescence assay and ANS displacement experiments, to determine the inhibitory activities against the amyloid fibril formation of TTR. In addition, some selected compounds were crystallised in complex with TTR in order to analyse the correlation between the binding mode of these ligands and their inhibitory activity^56^. The study revealed that CAPE, phenetyl ferulate and rosmarinic acid are the most potent derivatives among the CAPE and CA (caffeic acid) alkyl esters. The methylation of 3-OH can be tolerated and increases the inhibitory activity, while the methylation of 4-OH decreases it (Figure 10). The methylation of both 3-OH and 4-OH of CAPE is detrimental for the inhibitory activity. The NDGA and the dihydroguaiaretic acid (lignans) have also been tested by ThT assay, and demonstrated to inhibit fibril amyloid formation even better than the CAPE derivatives (Figure 10).

**Figure 11.**
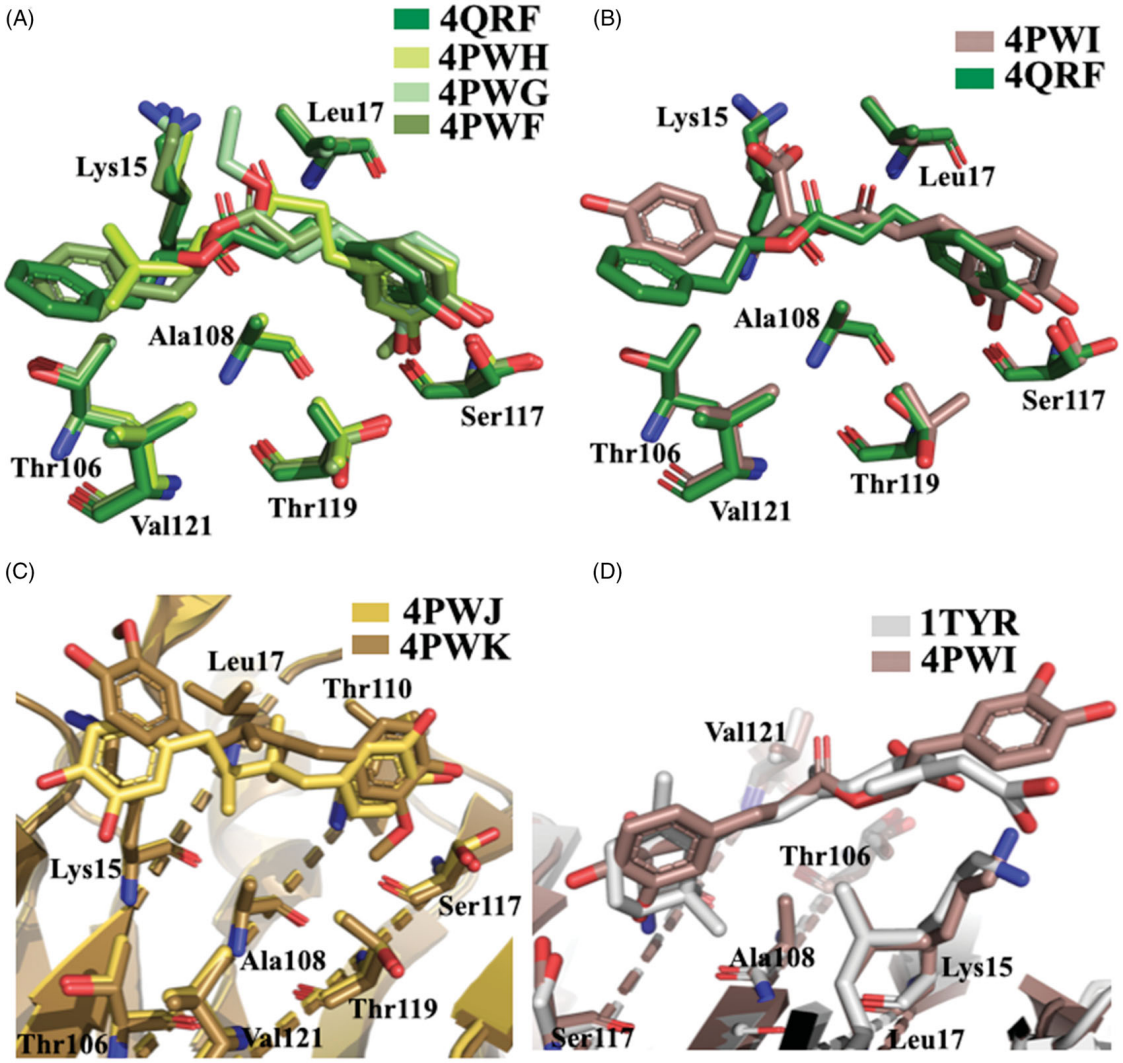
Structure analysis of caffeic acid, its derivatives, NDGA, and dihydroguaiaretic acid. (A) Superposition of V30MTTR-CAPE (4QRF), V30MTTR-1,1-dimethylallyl caffeate (4PWH), V30MTTR-ethyl caffeate (4PWG), and V30MTTR-phenethyl ferulate (4PWF) crystal complexes. (B) Comparison between V30MTTR-CAPE and V30MTTR-rosmarinic acid (4PWI) crystal complexes. (C) Superposition between V30MTTR-NDGA (4PWJ) and V30MTTR-dihydroguaiaretic acid (4PWK). (D) Superposition of V30MTTR-rosmarinic acid and TTR-retinoic acid (1TYR).

The crystal structures of caffeic acid, NDGA, and their derivatives, in complex with TTR V30M, were solved (4QRF, 4PWF, 4PWG, 4PWH, 4PWI, 4PWK, and 4PWJ, Table 1)^56^. The r.m.s.d. value calculated on Cα atoms between apo-V30M and V30M-compound complexes was in range from 0.30 to 0.56 Å, therefore, the ligand interaction with the T_4_-BS does not induced relevant structural differences. Regarding the caffeic acid derivatives, the catechol moieties of CAPE (4QRF), CA alkyl acids (4PWG and 4PWH), and the guaiacol function were located into the inner subsites, while the alkyl ether groups point towards the outer BS (Figure 11(A)). The oxycarbonyl and alkyl moieties of CA ethyl esters (ethyl caffeate and 1,1-dimethylallyl caffeate) are stabilised by hydrophobic interaction with amino acid side chain Lys15, Leu17, Thr106, Ala108, and Val121. Rosmarinic acid (4PWI) binds to TTR displacing similar orientation with one catechol function placed into the inner BS and the other pointing towards the outer cavity (Figure 11(B)). Rosmarinic acid interacts with TTR keeping the same interactions of the other CAPE and CA derivatives and in addition its carboxylic moiety makes salts bridge with Lys15 increasing the complex stability. The binding mode described for caffeic acid derivatives is analogue to that of NDGA (4PWJ) and its derivative dihydroguaiaretic acid (4PWK) (Figure 11(C)): one catechol group is located into the inner cavity and the other points towards the outer subsite, while the hydrophobic linker is contorted by a-polar side chain of Lys15, Leu17, Thr106, Ala108, and Val121. It is interesting to underline that only the dihydroguaiaretic acid derivative binds to the two TTR subunits in asymmetric way leading to the loss of the HB with Ser117 in the dimer BB’. As observed above, the 3-OCH_3_ of NDGA resulted as the most effective functional group for the inhibitory activity. The methoxy group is in fact, involved in hydrophobic interactions with Ala108, Leu110, Ser117, and Thr119 and hydrogen-bonded with Ser117. This observation confirms the importance of hydrophobicity to well interact with the inner cavity of the binding pocket and the necessity of HB donor/acceptor groups for the interaction with Ser117. A two-aromatic-ring substructure having a linker connecting them, such as biphenyl, stilbene, and biphenyl ether, seems to be a good template for the design of TTR amyloid fibril inhibitors. The linker length should be sufficient to enable the inhibitor to interact with the outer binding pocket, where other hydrophobic interactions with Thr106 and Val121 are necessary for a better affinity, and to reach the Lys15 for establish a salt bridge.

The chemical structure of rosmarinic acid recalls that of retinoic acid, one of the endogen TTR ligand. As mentioned above, retinoic acid can be transported by TTR through the interaction with RBP^21^. Moreover, a competition between the retinoid and T_4_ suggested that the ligands bind to the same sites, into the central channel of TTR tetramer^107^. The crystal structure of TTR in complex with retinoic acid was solved more than 20 years ago (1TYR)^57^. Retinoic acid binds to TTR fitting T_4_-BS with a negative cooperative mechanism. The orientation of retinoic acid into the T_4_-BS displays a similar interaction network as rosmarinic acid (Figure 11(D)). The cyclohexene ring is oriented in the inner pocket facing the Ser117 with C2 and C3, the two methyl groups interact with Thr119, Ala108, and Leu17, and the π-conjugated linker points towards the molecular surface (Val121) with the carboxylic acid that interacts with Lys15 (Figure 11(D)).

## Non-flavonoid polyphenol: resveratrol and curcumin

**Figure 12.**
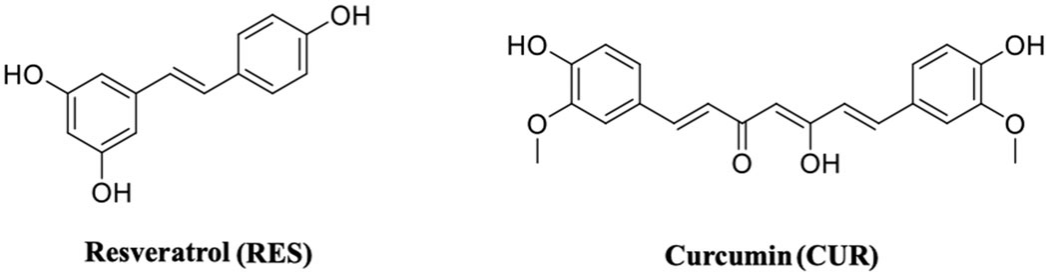
Molecular structures of resveratrol and curcumin.

**Resveratrol** (3,5,4′-trihydroxy-trans-stilbene) (**RES**) ( (Figure 12)) is a stilbenoid, a type of natural phenol, and a phytoalexin produced by several plants in response to injury or when the plant is under attack by pathogens, such as bacteria or fungi. Sources of RES in food include the skin of grapes, blueberries, raspberries, mulberries, and peanuts^108^. Its molecular structure shares the common architecture of CAPE analogues, with two aromatic rings separated by a linker. RES is an antioxidant, anti-inflammatory, and anticancer compoun^87^^,^^109^ and it has potential beneficial activities in the prevention of neurodegenerative diseases (Alzheimer’s disease, amyotrophic lateral sclerosis, Parkinson’s disease, and Huntington’s disease)^110^^,^^111^, including ATTR^58^. It has been reported that the neuroprotection effect of RES preserves the functionality of neuronal cell^112^. As previously mentioned for naringenin, also RES, in neuronal cells, increases the level of neuroglobin, a neuroprotective protein discovered in 2000^87^.

RES as well as the other polyphenols displays very low bioavailability due to their rapid metabolic degradation, therefore, not only RES but also its major metabolites were investigated in the study of TTR tetramer stabilisation.

**Figure 13.**
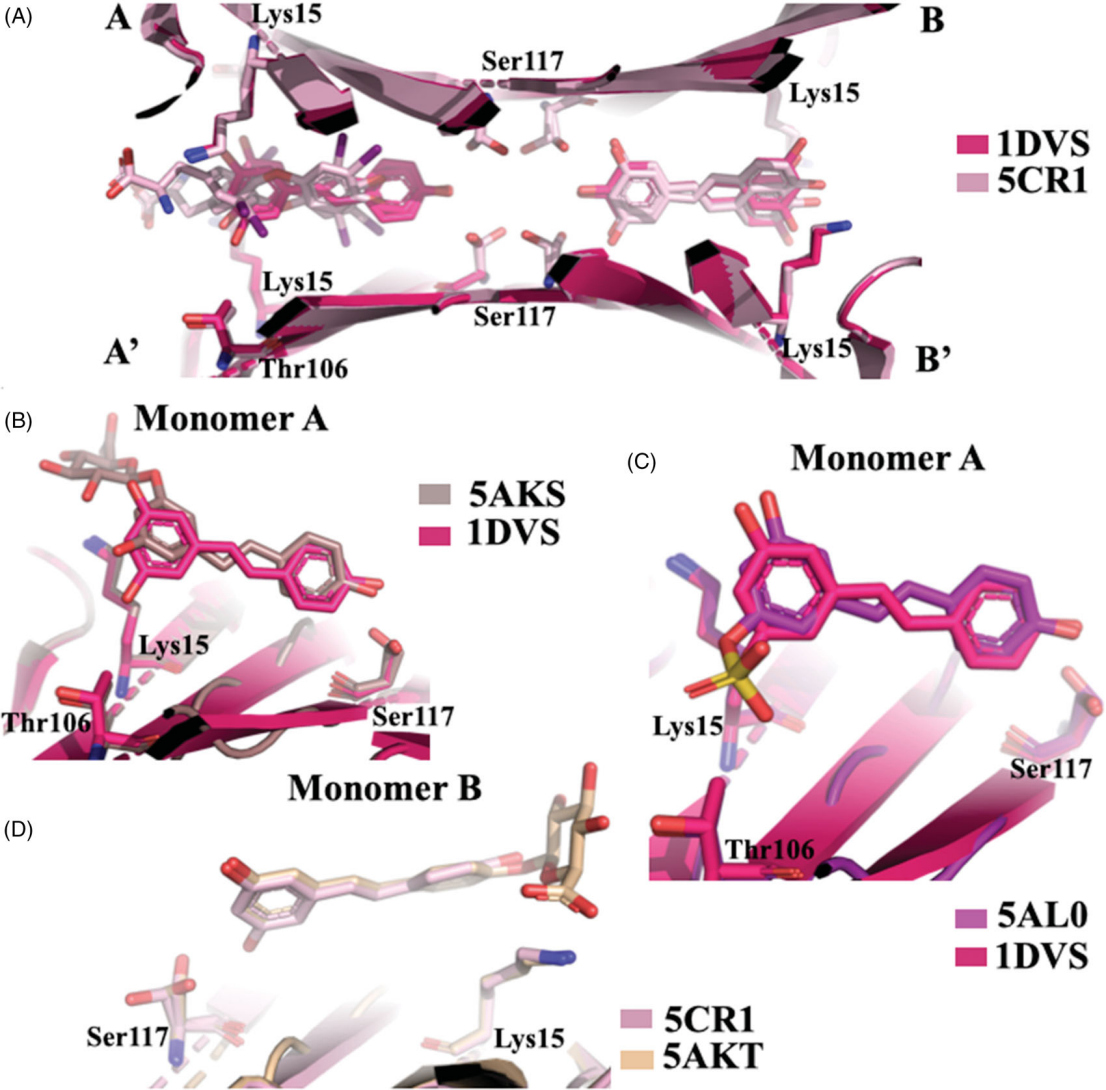
Structure analysis of resveratrol and its metabolic derivatives. (A) Superposition between the two crystal structures TTR-RES deposited at PDB (1DVS *versus* 5CR1). (B) Comparison between TTR-3-O-glucuronide complex (5AKS) and TTR-RES (1DVS). (C) Superposition of TTR-4’-O-glucuronide crystal complex (5AKT) and TTR-RES (5CR1). (D) Superposition of TTR-3-O-sulphate complex (5AL0) and TTR-RES (1DVS).

RES showed to be able to have a binding affinity for TTR by fluorimetric binding assay, while the affinities of its metabolites (RES 3-O sulphate, RES 3-O-glucoronide, and RES 4′-glucorinide) resulted lower^52^. However, by the same assay, T_4_ did not effectively compete with bound RES, even if an interaction of T_4_ with TTR has been observed. Competition binding assays in the presence of radiolabeled T_4_ revealed that RES was not able to displace the bound T_4_. The stabilising effect against urea denaturation and the inhibition activity against TTR fibrillogenesis assessed by turbidimetric assay of RES and its metabolite resveratrol-3-O-sulphate, were found to be significantly similar to polyphenols^52^. The first TTR-RES crystal complex was deposited at the PDB data bank in 2000 (1DVS code, Table 1)^58^, while in 2015 a second structure in which TTR was crystallised with both RES and T_4_ was solved (5CR1)^52^. The presence of T_4_ molecule is relatively easy to detect because, recording the anomalous signal given by iodine atoms, the high density picks unambiguously correspond to the iodine position. Using this strategy, the presence of the two ligands was investigated and the RES molecule was present essentially in the dimer B/B’ while T_4_ was located mostly in pocket A/A’. This result suggested that RES is characterised by negative cooperativity binding mode as already observed for several TTR ligands. The density map shows that the 3,5-dihydroxyphenyl portion is placed in the inner binding pocket establishing an HB with Ser117, so the orientation of RES is opposite to that described in the first deposited structure (1DVS) (Figure 13(A)). A plausible explanation was attributed to the different crystallisation protocols applied between the two laboratories: the structure 1DVS was obtained soaking the ligand into the TTR empty crystal while the other, PDB code 5CR1, was prepared by co-crystallisation^52^.

In order to investigate the binding mode of the major RES metabolites, the crystal structures of TTR in complex with RES 3 and 4′ O-glucuronide and 3-O-sulphate were solved^52^. The analysis of TTR in complex with RES 3-O-glucuronide showed that the ligand binds to T_4_-BS with the same of the first deposited TTR-RES complex 1DVS, with the monohydroxyphenyl group located into the inner cavity and the glucuronide portion pointing towards outside of the pocket (Figure 13(B)). The same binding mode is also observed for RES 3-O-sulphate (Figure 13(C)), while an opposite binding mode is displaced by 4′ O-glucuronide (Figure 13(D)). It is interesting underline that the binding affinities of glucuronides derivatives and the capability to stabilise the TTR tetramer decrease in comparison with the aglycon RES, except for 3-O-sulphate that stabilise the TTR tetramer by the interaction between the sulphate group and the Lys15.

**Curcumin** (**CUR**) is a compound from *Curcuma longa*, a member of the ginger family (Figure 12). Chemically, CUR is a diarylheptanoid, belonging to the group of curcuminoids, which are natural phenols responsible for turmeric’s yellow colour. CUR has been studied for its anti-inflammatory effects and its potential activity against cardiovascular, pulmonary, autoimmune, neoplastic, and neurodegenerative diseases (Alzheimer’s, Parkinson’s, and Huntington’s diseases)^113^^,^^114^. It has been shown to be of therapeutic value in preventing the formation and extension of β-amyloid fibrils^115^. For the first time, in 2009, CUR was described as a molecule capable to bind and stabilise the TTR tetramer^116^. The binding affinity of CUR for wt-TTR has been established using Scatchard analysis of fluorescence quenching (*K*_d_=2.3 × 1 0 ^−6 ^M). In the same study, with the aim to understand whether CUR binds to the T_4_-BS, the ANS-displacement assay was used. CUR decreased the ANS fluorescence suggesting that CUR binds into the TTR T_4_-BS^116^. Since then, several studies focussed on TTR-CUR interaction have been done and, in 2019, an exhaustive review, that summarise all these researches, was written by Ferreira et al.^92^. Despite the proof of binding, CUR is not able to prevent the acid induced aggregation of TTR, probably because in the test conditions CUR undergoes protonation and isomerisation of the phenolic and enolic hydroxyl groups which might impair the interaction that is normally established at physiological pH. TEM and DLS assays, in fact, showed the ability of CUR to redirect the TTR amyloid formation pathway into a monodispersed population of “off-pathway” oligomers which resulted less toxic than “on-pathway” aggregate intermediates^91^.

*In vivo* studies with CUR revealed that CUR reduces TTR load and degrades amyloid deposits in tissues^117^, by increasing the TTR tetramer resistance to dissociation as previously observed *in vitro,* by IEF studies in semi-denaturation conditions.

**Figure 14.**
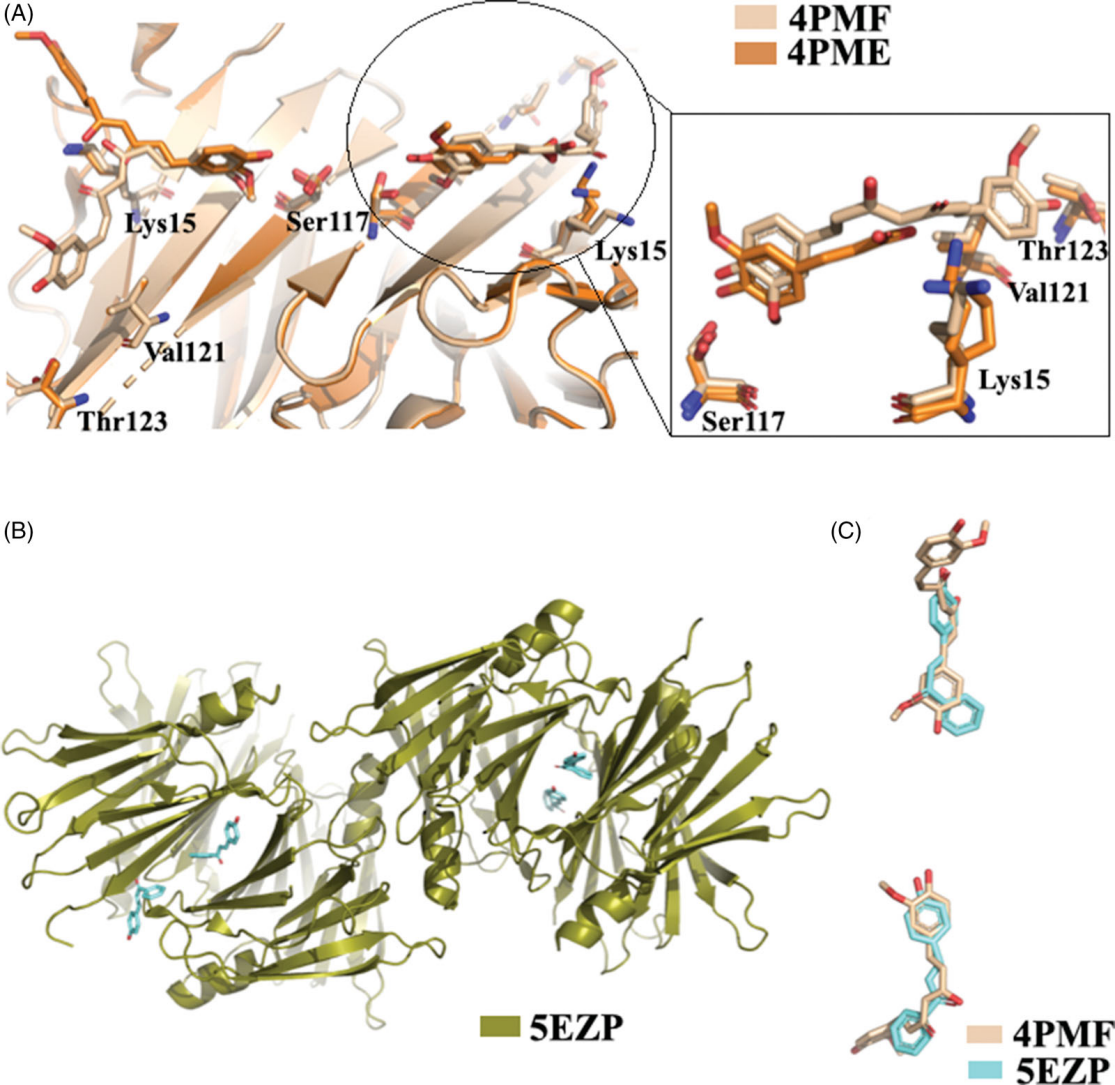
Structural analysis of TTR in complex with CUR, ferulic acid, and 4-hydroxychalcone derivatives. (A) Superposition of the two different TTR-CUR crystal complex 4PMF *versus* 4PME. (B) Asymmetric unit of trigonal TTR crystal in complex with hydroxychalcone derivative. (C) Comparison between curcumin and 4-hydroxychalcone ligand.

The X-ray analysis of TTR-CUR crystal complex (4PMF)^59^ confirmed the binging mode previously predicted by ANS-displacement assay^116^. The CUR molecules bind to the two T_4_-BS sites on TTR in a slightly different manner^59^. Six TTR-CUR co-crystals have been collected, and in one of those the presence of CUR degradation product was detected (4PME). All crystals of TTR-CUR complex grew belong to the orthorhombic space group P2_1_2_1_2 and the typical TTR tetramer structure is preserved. The CUR ligands fully occupy the T_4_-BS (AA’/BB’) from Ser117 in the inner cavity to Lys15, at the entrance of the BS. In addition, CUR stretches beyond Val121 and Thr123 (Figure 14(A)). The structure analysis of another TTR-CUR crystal complex (4PME), clearly showed that in binding site A/A’ the electron density map corresponds to CUR, while the second binding pocket B/B’ is occupied by ferulic acid, a CUR degradation product. The aromatic ring of ferulic acid is located into the inner binding pocket and the carboxylic group is oriented towards Lys15^59^.

The biological and structural investigation between TTR and CUR suggested that some structural elements are important for the affinity of CUR with the target: the two-aromatic rings architecture, the presence of an unsaturated linker between the two aromatic rings with variable length, the methoxy and hydroxyl group in positions 3 and 4 of the aromatic moiety and an eventual carboxylic group able to form a salt bridge.

CUR in not very soluble in water solution and rapidly degrades in several chemical species, for this reason one approach is to study CUR-like compounds. In order to discover new CUR-like ligands, the TTR-4-hydroxychalcone crystal complex was solved^60^. To protect both the ligand and the protein from radiation damage occurring during data collection, the helicoidal scan method was used to record the data^118^. The crystal complex grown at pH 8.5 (5EZP) showed a new TTR polymorph: trigonal space group P3_1_ (Figure 14(B)). The crystal grown in space group P3_1_ does not suffer from the characteristic problem of 2-fold ligand density averaging due to the orthorhombic P2_1_2_1_2 space-group, in which the majority of TTR ligand complexes have been solved. The absence of any 2-fold crystallographic symmetry operation facilitates the positioning of the inhibitor and it eliminates the positional uncertainty derived from placing an asymmetric ligand in a site at a dimer interface. A comparison between CUR and 4-hydroxychalcone displayed that they similarly bind to TTR^60^ (Figure 14(C)).

CUR, as other natural compounds, remains a promising scaffold for the development of potent multi-stage disease-modifying drugs for the treatment of TTR amyloidosis.

## Conclusion

Today the use of natural compounds and plant extracts, normally taken with the diet, represents a potential therapeutic approach against several pathologies. In the last years, natural compounds have largely been studied for their promising neuroprotective effects against several neurodegenerative diseases such as Alzheimer’s, Parkinson’s, Huntington’s diseases, amyotrophic lateral sclerosis, and multiple sclerosis. Most of these neurodegenerative disorders can be ascribed to amyloidogenic proteins, such as TTR, going to self-assemble into toxic insoluble aggregates. TTR deposits most frequently occur in the peripheral nervous system, resulting in peripheral neuropathy.

The literature examination here reported gives a temporal view of the studies performed on natural compounds as inhibitors of TTR amyloidosis. In this review, we discussed the chemical and structural data of natural compounds proposing for each class of compounds a structure-activity relationship that can provide a pipeline of different scaffolds useful in the design and optimisation of new drugs against TTR amyloidosis.

Concluding, natural products can be considered promising therapeutic agents for the prevention of TTR fibrillisation.
